# Longitudinal Monitoring of Alpha-Fetoprotein by Dried Blood Spot for Hepatoblastoma Screening in Beckwith–Wiedemann Syndrome

**DOI:** 10.3390/cancers11010086

**Published:** 2019-01-14

**Authors:** Alessandro Mussa, Valentina Pia Ciuffreda, Pina Sauro, Veronica Pagliardini, Severo Pagliardini, Diana Carli, Jennifer M. Kalish, Franca Fagioli, Enza Pavanello, Giovanni Battista Ferrero

**Affiliations:** 1 Department of Public Health and Pediatric Sciences, University of Torino, 10126 Torino, Italy; valentina.ciuffreda@edu.unito.it (V.P.C.); pina.sauro@edu.unito.it (P.S.); veronica.pagliardini@libero.it (V.P.); pagliardini.severo@virgilio.it (S.P.); diana.carli@unito.it (D.C.); franca.fagioli@unito.it (F.F.); epavanello@cittadellasalute.to.it (E.P.); giovannibattista.ferrero@unito.it (G.B.F.); 2 Neonatal Intensive Care Unit, Department of Obstetrics and Gynecology, S.Anna Hospital, Città della Salute e della Scienza, 10126 Torino, Italy; 3 Division of Human Genetics, the Children’s Hospital of Philadelphia and Department of Pediatrics, The Perelman School of Medicine, University of Pennsylvania, Philadelphia, PA 19104, USA; kalishj@email.chop.edu; 4 Pediatric Onco-Hematology, Stem Cell Transplantation and Cellular Therapy Division, Regina Margherita Children’s Hospital, Città della Salute e della Scienza, 10126 Torino, Italy

**Keywords:** alpha-fetoprotein, Beckwith–Wiedemann syndrome, dried blood spot, hepatoblastoma, screening

## Abstract

Background: Hepatoblastoma screening in the Beckwith–Wiedemann spectrum (BWSp) is currently based on measuring a specific serum marker alpha-fetoprotein (αFP) every three months until the fourth birthday. Frequent blood draws can be a burden for patients and their families. Methods: We have developed a less invasive alternative testing method based on measuring αFPs from dried blood spots (DBS). The method was validated with 259 simultaneous plasma and DBS αFP measurements in 171 children (132 controls and 39 patients with BWSp). Results: The DBS and plasma measurements overlapped across the wide range of αFP concentrations independent of patient age (*p* < 0.0001), demonstrating the utility of this method for longitudinal monitoring. Occasional differences between measurements by the two techniques fell within standard laboratory error and would not alter clinical management. Conclusions: This novel method shows consistent overlap with the traditional blood draws, thereby demonstrating its utility for hepatoblastoma screening in this setting and alleviating the burden of frequent blood draws. This also may help increase patient compliance and reduce costs of health care screening. The DBS-based method for the measurement of cancer biomarkers may also be applied to several other chronic diseases with increased risks of αFP-producing liver tumors.

## 1. Introduction

The Beckwith–Wiedemann spectrum (BWSp) consists of the variable association of macroglossia, abdominal wall defects, organomegaly, ear pits/creases, facial nevus simplex, hyperinsulinemic hypoglycemia, lateralized overgrowth, and embryonal tumor predisposition [1,2,3]. BWSp includes the classical Beckwith–Wiedemann syndrome (BWS, OMIM #130650), the most common overgrowth and cancer predisposition disorder (1:10,500 live births) [4], and more subtle presentations with an 11p15.5 molecular anomaly, including Isolated Lateralized Overgrowth (ILO, OMIM #235000) [5]. The BWSp embryonal tumor predisposition in childhood includes an increased risk of developing hepatoblastoma (HB), which occurs in up to 3.5% of patients depending on the specific genetic anomaly [6,7]. HB typically occurs before 30 months of age with a peak of incidence at six months [8,9], and BWS represents the most relevant risk factor for HB, with a relative risk of 2280 times greater than in the general population [10]. The molecular subgroups of BWS patients with the highest risk for HB are those with paternal uniparental disomy (UPD) of chromosome 11 or genome-wide UPD [11,12,13]. Moreover, HB is the most common tumor diagnosed in BWSp children with the loss of methylation at imprinting control region 2, the molecular subgroup representing approximately 50% of BWSp patients [14]. 

HB usually grows rapidly; therefore, survival and prognosis are highly dependent on early diagnosis [8,9,15]. More than 95% of HB secrete the highly sensitive and specific tumor marker alpha-fetoprotein (αFP) [9,15], and measuring αFPs are used in diagnosis, follow-up, and relapse detection. BWSp patients are monitored for HB with αFPs every three months from birth to the fourth birthday [16,17,18,19]. Screening provides the opportunity for early detection and at earlier stages of the diagnosis, potentially allowing for less toxic therapies [20,21]. 

However, αFP screening in this population has been controversial for a number of reasons [6,7,14,22,23,24]. First, the variability in the physiologic decrease of normal serum αFP levels (from 10^5^ U/mL magnitude at birth to concentrations steadily <10 U/mL by the age of 12–24 months) leads to challenges in the interpretation of αFP levels in early infancy [25,26]. Moreover, normal αFP values in BWS patients in the first year of life tend to be elevated compared with normal pediatric values [27]. Age-corrected reference values should be employed [25,26], and αFP trends, rather than reliance of the actual value compared to non-BWS norms, is a more accurate screening strategy [16,23,28].

Second, some health care providers consider the incidence of HB too low to warrant specific screening [2]: While recently the American Association for Cancer Research (AACR) Childhood Cancer Predisposition Workshop adopted a 1% risk threshold for surveillance and recom­mended αFP screening for all cases of BWSp [29], a consensus statement from the European Network of Human Congenital Imprinting Disorders (EUCID) judged a 5% threshold to be more appropriate for European healthcare systems and did not recommend αFP screening in these patients [2].

Lastly, the frequent blood draw schedule in early infancy is perceived as invasive, represents a burden for some children and families, and may cause compliance issues [22,23]. However, parents report being comforted and reassured by the screening [30]. For this reason, in a previous report we demonstrated the technical feasibility of αFP determination using dried capillary blood spots (DBS) [31]. Here we demonstrate the utility of DBS in parallel to the currently accepted practice of venous αFP sampling in a range of BWSp patients and normal controls. The method described in our previous report was introduced as a clinical practice pilot program in our institution and compared in parallel to the standard laboratory method. In this report, we describe our experience in the longitudinal monitoring of BWSp by the novel method, further supporting its utility in routine clinical practice. 

## 2. Results

**Figure 1:**
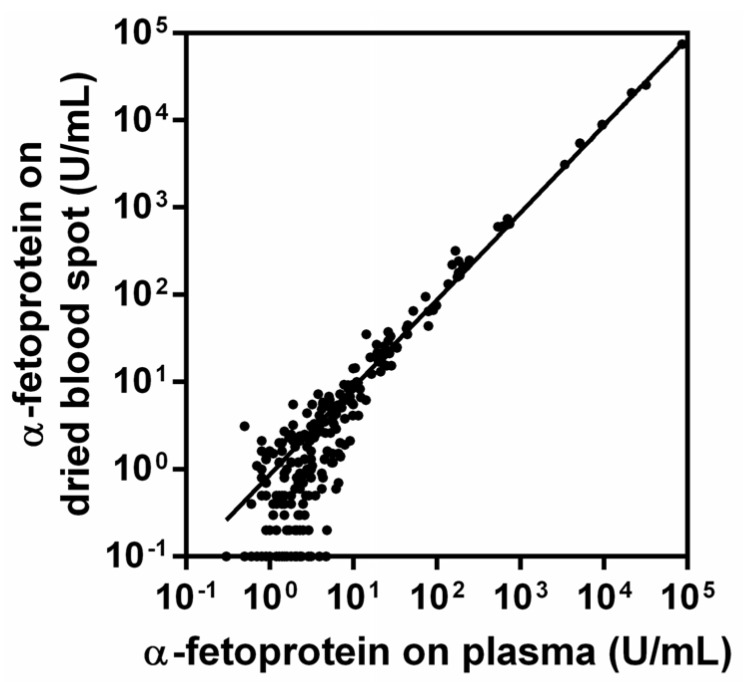
Correlation between α-fetoprotein (αPF) measured on plasma and on dried blood spot (DBS) (*r*^2^ = 0.999, *p* < 0.001).

Measurements on plasma and DBS were closely correlated (*r*^2^ = 0.999, *p* < 0.001, Figure 1). Raw measurement data are provided in Supplementary Table S1. The αFP measurements by the two methods showed consistency and largely overlapped across the wide range of physiological concentrations of the tumor marker (0.3–97,198.0 U/mL in plasma and from 0.1 to 97,889.0 U/mL on DBS). 

In 202 cases, both methods measured αFP ≤10 U/mL, whereas in 51 cases, both measurements were >10 U/mL. In five cases, αFP was >10 U/mL in plasma and ≤10 U/mL on DBS: Patient 3 with 11.4 U/mL on plasma and 9.8 U/mL on DBS, Patient 4 with 11.5 U/mL on plasma and 4.1 U/mL on DBS, and Patient 5 with 12.0 U/mL on plasma and 8.3 U/mL on DBS. In one case, serum αFP was ≤10 U/mL (9.9 U/mL) and >10 U/mL on DBS (14.3 U/mL). All the paired measurements show differences between serum and DBS measurements within the coefficient of variation (CV%). Twenty-six patients and 20 controls had more than one αFP measurement; 22 patients and 123 controls had 1 paired measurement. 

**Figure 2:**
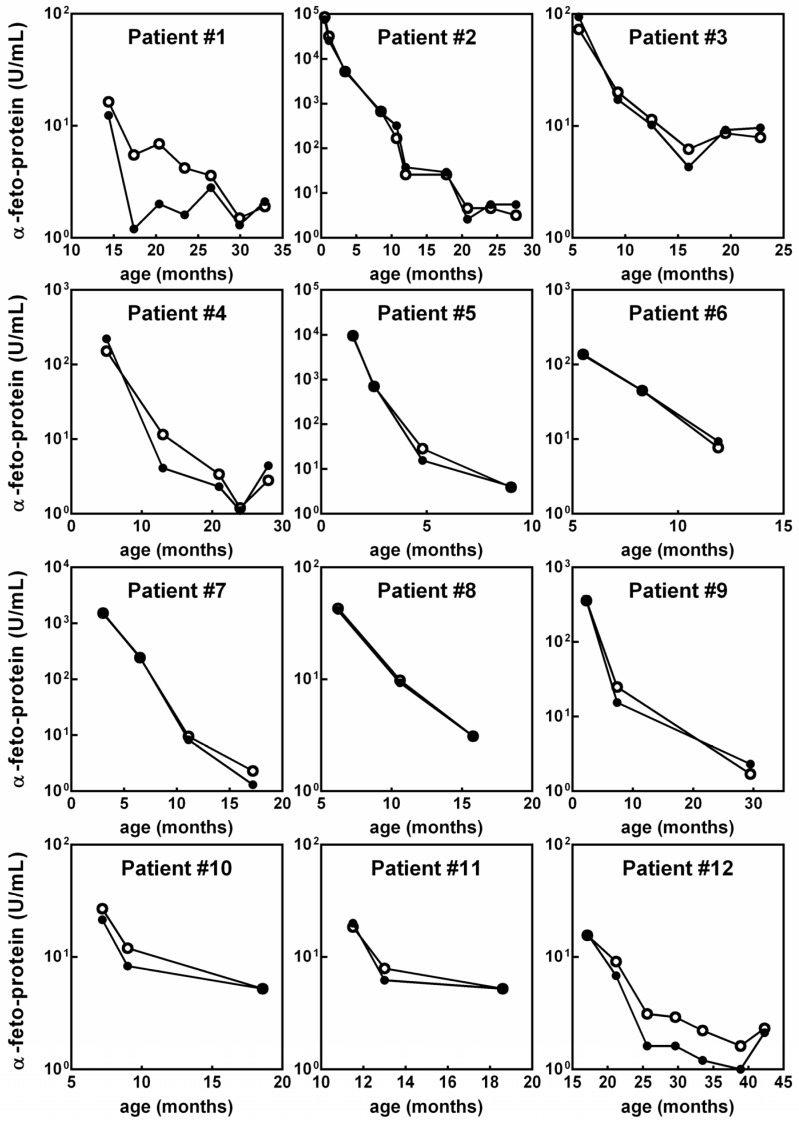
The longitudinal evaluation of alpha-fetoprotein (αFP) plasmatic concentration on dried blood spots (DBS, closed circles) overlapped that on standard laboratory method (open circles) in the 12 patients affected by cancer-predisposition syndromes with αFP concentrations >10 U/mL.

Of the 26 patients with a longitudinal assessment, 12 patients had αFP measurements >10 U/mL: Their αFP trend over time is displayed in Figure 2 to highlight the concordance between the two methods. The remaining 14 patients were assessed longitudinally, and all had concordant DBS-plasma αFP <10 U/mL over time (not shown as values under 10 U/mL are considered within normal range). Of the 22 patients tested with a single paired measurement, 19 had concordant DBS-plasma αFP values <10 U/mL and three had values >10 U/mL. In the latter group, Patient 29 showed 225.0 U/mL in plasma and 212.0 U/mL on DBS, Patient 30 had 609.0 U/mL in plasma and 610.5 U/mL on DBS, and Patient 37 had 187.1 U/mL in plasma and 168.0 U/mL on DBS. The patient diagnosed with non-syndromic HB, a female aged 31 months, had an αFP of 583.5 U/mL measured by the traditional method and 601.0 U/mL on DBS.

## 3. Discussion

In this work, we demonstrate the feasibility of HB screening in overgrowth-cancer predisposition syndromes using DBS for αFP measurements. DBS, a novel method to measure αFP concentration showed consistent overlap with the traditional venous sampling method, showing reliability in the clinical setting of a tumor screening program. In the first two years of life—those in which the likelihood of developing HB is commonly higher in such conditions [9]—αFP concentrations decrease rapidly from a 10^6^–10^5^ to a <10 U/mL magnitude, with almost unpredictable and variable timing, making interpretations challenging. Although age-specific and gestational age-corrected normal αFP concentrations values are available [32], HB screening relies mostly on the longitudinal observation of repeated measurements of αFP concentrations rather than on the detection of a single measurement [28,29]. The DBS measurements overlapped the serum measurements across a wide range of physiologic concentrations and ages, demonstrating the utility of our methodology for longitudinal monitoring in both newborns and toddlers. Moreover, the range of measurements includes a 10^5^ magnitude often observed in prematurity [32], which is common in the BWSp [33].

After the physiological decrease of αFP serum concentrations, a cutoff of >10 U/mL is commonly used to define normal values after 2 years of age. The measurement provided by the traditional and novel methods consistently matched across this diagnostic threshold, allowing, therefore, the determination of normal screens compared to abnormal screening tests. The few cases with discordant plasma and DBS measurements showed very tight fluctuations across the 10 U/mL threshold within the acceptable error range for these tests, and clinical management was not altered by these fluctuations. 

Additionally, our patient who was ultimately diagnosed with a non-syndromic HB, showed that αFP levels determined by the two methods were highly consistent in the setting of a tumor diagnosis as well. Hence, we propose using DBS for HB screening and for patients presenting abnormal results suggesting a tumor diagnosis; further investigation with both conventional venous αFP testing and imaging studies should be used to confirm or exclude a HB diagnosis. Finally, the DBS technique alleviates the burden of frequent blood draws and is simple, efficient and low-cost, thus making the routine measurement of αFP more practical. These aspects are crucial to improve patients’ compliance with tumor surveillance recommendations for cancer predisposition syndromes. It has recently been shown that accurate knowledge about cancer risk and screening in the BWSp context decreases parents’ worries about tumor development [30] and that the DBS method may also decrease children’s anxiety related to HB screening. The DBS method is cheaper in terms of storage, transport, and handling even compared to other minimally invasive methods (i.e., finger poke in microtainer tube) that require that the samples remain in a liquid state [34]. DBS home sampling is associated with a reduction in costs both from a healthcare and from a societal perspective with patient costs abated nearly to zero and with a relevant decrease in costs related to the loss of productivity [35].

Other potential applications of the DBS method may come from this work and include screening of other conditions with increased risk of hepatocarcinoma or other αFP-secreting tumors as well as follow-up of patients treated for liver tumors. As screening for hepatocarcinoma in cirrhotic patients by repeated serum αFP measurement is feasible [36] and results in increased survival rates [28,37], monitoring in cirrhotic patients by DBS may represent a specific example of future applications. 

## 4. Materials and Methods

### 4.1. Patients

Overall, 259 simultaneous plasma and DBS αFP measurements were performed in 171 children (mean age 38.7 ± 59.2 months, range 0–7.3 years, 88 males, 83 females). Of these, 116 paired measurements have been performed in 48 patients with syndromes with increased risk of HB for tumor screening (range 0–60 months): 39 were affected by BWSp/ILO (23 molecularly confirmed, 16 diagnosed clinically with negative molecular tests), 3 had macrocephaly-capillary malformation syndrome, 5 had undiagnosed likely syndromic overgrowth disorders, and one girl had an isolated HB. Of these, 31 measurements from 31 patients were previously reported in our preliminary report [31]. The remaining 143 paired measurements were performed in 123 children as controls: 27 were healthy children and 96 underwent blood tests for suspected conditions with no effect on plasmatic αFP concentration (20 suspected or well compensated thyroid disorders, 35 with recently healed infections, 12 serum lipids screening, 13 affected by phenylketonuria, 10 suspected iron deficient anemia, and 6 suspected precocious puberty). Besides studying the potential utility of the DBS method for longitudinal monitoring, we applied the novel method to patients admitted for suspected abdominal tumors. Twenty-three paired measurements were therefore performed in patients referred to our Division of Pediatric Oncology for suspected neoplasms and who had αFP measurement with the aim to identify potential αFP producing tumors. Among the last cases, the suspected diagnosis of neoplasm was excluded after the tests (including αFP and appropriate imaging) except for one female who was ultimately diagnosed with non-syndromic HB. 

### 4.2. Study and Screening Protocol

Informed consent was obtained from parents using a study protocol approved by the Institutional Review Board of our University Hospital (IRB number CS/156/2014 Città della Salute e della Scienza di Torino, University of Torino, Italy). Our protocol for HB screening in overgrowth-cancer predisposition syndromes is consistent with that proposed by the American Association for Cancer Research [29] and is based on 3 months’ abdominal ultrasound and simultaneous αFP measurements by standard laboratory methodology from birth up to the fourth birthday [17]. A negative αFP test is defined as an αFP measurement less than 10 U/mL (1 U/mL = 1.21 ng/mL) or declining with respect to previous measurement. The individual value is interpreted in the context of the αFP trend over time, with an expectation of declining values through infancy. In case of concentrations >10 U/mL, the results need to be interpreted on the basis of normal BWS values (which tend to be elevated over the first years of life compared with normal pediatric values), with age-specific and gestational age-corrected reference values provided in the literature [25] and with previous measurements performed in the same patient, if available. If the concentrations are less than the previous ones, then we consider the test negative and register the absolute value in order to perform subsequent comparisons. If we detect an αFP greater than the previous one, the test is referred to as positive, recent imaging is re-evaluated, and the patient is recalled for subsequent αFP remeasurements after a 6-week interval for rises greater than 50–100 U/mL [29]. In cases of significantly larger increases (greater than 1000 U/mL) or with further increase at the 6 weeks αFP remeasurement, second-step medical investigations are proposed (targeted liver US or MRI) [29]. 

### 4.3. Laboratory Assays

Paired αFP measurements were simultaneously performed on blood, obtained by venipuncture, and DBS, collected by heel-stick or by spotting single blood drops from a syringe directly onto standard filter paper employed for newborn screening. The DBS specimens were dried at room temperature, routinely stored in plastic bags at 4 °C and analyzed employing a 3.2 mm-diameter spotted filter paper punch containing approximately 3.4 µL of adsorbed blood. The serum αFP measurement kit (AutoDELFIA hAFP, Perkin Elmer, Waltham, Massachusetts) adapted to the DBS technique has been employed, as previously described [31]. The intra-assay CV% was evaluated for quality controls: For low concentrations (~10 U/mL), the CV%s were 3.02% for the plasma assay and 4.11% for the DBS one; for high concentrations quality controls (~70 U/mL), the CV%s were 3.05% and 3.22%, respectively. 

### 4.4. Statistics

Data were analysed by GraphPad Prism 6.0 (GraphPad Software, Inc. La Jolla, CA, USA). Data distribution was assessed by the Shapiro–Wilk test and correlations tested by Pearson or Sperman methods, accordingly. 

## 5. Conclusions

In conclusion, in this study we demonstrate that screening children with overgrowth disorders with HB predispositions can be performed by a novel simple method, which measures αPF on DBS. This novel technique may lead to increased adherence and reduced anxiety and cost. 

## Supplementary Materials

The following are available online at http://www.mdpi.com/2072-6694/11/1/86/s1, Table S1: Raw data of the paired measurements of alpha-fetoprotein (αFP) with the traditional and dried blood spot (DBS) methods.
