# Supplementary material for: Longitudinal Monitoring of Alpha-Fetoprotein by Dried Blood Spot for Hepatoblastoma Screening in Beckwith–Wiedemann Syndrome
Source: Cancers (Basel). 2019 Jan 14;11(1):86. doi: 10.3390/cancers11010086 (PMC6356556; doi:10.3390/cancers11010086)
Supplement: Supplementary file 1 [file cancers-11-00086-s001.pdf]

Supplementary Material

Longitudinal Monitoring of Alpha-Fetoprotein by Dried Blood Spot for Hepatoblastoma Screening in Beckwith–Wiedemann Syndrome

Alessandro Mussa, Valentina Pia Ciuffreda, Pina Sauro, Veronica Pagliardini, Severo Pagliardini, Diana Carli, Jennifer M. Kalish, Franca Fagioli, Enza Pavanello and Giovanni Battista Ferrero

Table S1. Raw data of paired measurements of alpha-fetoprotein (αFP) with traditional and dried blood spot (DBS) method.

Case ID	Patient ID	Condition/Diagnosis	Sample	Age (Months)	Plasma αFP	DBS αFP
1	HE1	Hepatoblastoma	1	31.4	538.0	601.0
2	UO1	Undiagnosed Overgrowth	1	21.5	4.5	1.3
3	UO1	Undiagnosed Overgrowth	2	29.5	5.3	2.6
4	BWS1	Beckwith-Wiedemann	1	14.4	16.4	12.4
5	BWS1	Beckwith-Wiedemann	2	17.4	5.5	1.2
6	BWS1	Beckwith-Wiedemann	3	20.4	6.9	2.0
7	BWS1	Beckwith-Wiedemann	4	23.4	4.2	0.6
8	BWS1	Beckwith-Wiedemann	5	26.5	3.6	2.8
9	BWS1	Beckwith-Wiedemann	6	29.9	1.5	0.9
10	BWS1	Beckwith-Wiedemann	7	32.9	1.9	2.1
11	BWS2	Beckwith-Wiedemann	1	102.6	0.7	0.1
12	ILO1	Isolated Lateralized Overgrowth	1	141.0	0.9	0.5
13	BWS3	Beckwith-Wiedemann	1	1.5	9544.0	8945.0
14	BWS3	Beckwith-Wiedemann	2	2.5	702.0	740.0
15	BWS3	Beckwith-Wiedemann	3	4.8	28.5	15.5
16	BWS3	Beckwith-Wiedemann	4	9.0	3.9	4.1
17	BWS4	Beckwith-Wiedemann	1	5.5	137.0	133.0
18	BWS4	Beckwith-Wiedemann	2	8.3	44.9	44.6
19	BWS4	Beckwith-Wiedemann	3	11.9	7.7	9.3
20	ILO2	Isolated Lateralized Overgrowth	1	110.3	1.8	0.4

21	BWS5	Beckwith-Wiedemann	1	152.9	2.2	0.7
22	UO2	Undiagnosed Overgrowth	2	52.8	0.6	0.4
23	UO2	Undiagnosed Overgrowth	1	41.1	1.4	0.1
24	MCM1	Macrocephaly-Capillary Malformation Syndrome	1	21.5	4.2	0.9
25	MCM1	Macrocephaly-Capillary Malformation Syndrome	2	25.6	2.7	0.9
26	BWS6	Beckwith-Wiedemann	1	81.3	5.7	1.2
27	ILO3	Isolated Lateralized Overgrowth	1	39.6	1.8	1.2
28	ILO3	Isolated Lateralized Overgrowth	2	47.4	1.3	1.2
29	ILO3	Isolated Lateralized Overgrowth	3	52.9	2.0	1.8
30	UO3	Undiagnosed Overgrowth	1	79.6	1.3	0.1
31	UO3	Undiagnosed Overgrowth	2	82.6	1.8	0.1
32	UO3	Undiagnosed Overgrowth	3	87.7	2.1	0.1
33	UO3	Undiagnosed Overgrowth	4	91.9	2.6	0.3
34	ILO4	Isolated Lateralized Overgrowth	1	14.6	6.6	1.5
35	BWS7	Beckwith-Wiedemann	1	4.6	152.0	222.0
36	BWS7	Beckwith-Wiedemann	2	13.0	11.5	4.1
37	BWS7	Beckwith-Wiedemann	3	21.1	3.4	2.3
38	BWS7	Beckwith-Wiedemann	4	23.9	1.2	0.5
39	BWS7	Beckwith-Wiedemann	5	27.6	2.8	4.4
40	BWS8	Beckwith-Wiedemann	1	138.3	2.1	2.2
41	BWS9	Beckwith-Wiedemann	1	55.0	1.7	0.1
42	BWS10	Beckwith-Wiedemann	1	67.6	1.9	5.5
43	MCM2	Macrocephaly-Capillary Malformation Syndrome	1	3.0	187.1	168.0
44	UO4	Undiagnosed Overgrowth	1	23.2	0.9	0.7
45	UO4	Undiagnosed Overgrowth	2	32.2	0.8	1.0
46	UO5	Undiagnosed Overgrowth	1	140.5	0.7	1.1
47	ILO5	Isolated Lateralized Overgrowth	1	159.9	0.8	1.6
48	BWS11	Beckwith-Wiedemann	1	0.5		
49	BWS11	Beckwith-Wiedemann	2	1.1	31812.0	25568.0
50	BWS11	Beckwith-Wiedemann	3	3.4	5179.0	5521.0
51	BWS11	Beckwith-Wiedemann	4	8.5	670.0	630.0

52	BWS11	Beckwith-Wiedemann	5	10.7	166.4	321.0
53	BWS11	Beckwith-Wiedemann	6	12.0	26.0	37.5
54	BWS11	Beckwith-Wiedemann	7	17.8	26.0	29.4
55	BWS11	Beckwith-Wiedemann	8	20.8	4.6	2.6
56	BWS11	Beckwith-Wiedemann	9	24.1	4.6	5.5
57	BWS11	Beckwith-Wiedemann	10	27.7	3.2	5.5
58	BWS12	Beckwith-Wiedemann	1	6.5		
59	BWS12	Beckwith-Wiedemann	2	11.1	9.5	8.3
60	BWS12	Beckwith-Wiedemann	3	17.2	2.3	0.3
61	MCM3	Macrocephaly-Capillary Malformation Syndrome	1	191.0	1.0	0.2
62	BWS13	Beckwith-Wiedemann	1	39.0	4.3	0.8
63	BWS13	Beckwith-Wiedemann	2	48.2	2.5	2.4
64	BWS13	Beckwith-Wiedemann	3	54.6	2.5	0.4
65	BWS14	Beckwith-Wiedemann	1	60.3	1.5	0.8
66	BWS15	Beckwith-Wiedemann	1	7.4	24.9	15.4
67	BWS15	Beckwith-Wiedemann	2	29.5	1.7	2.3
68	ILO6	Isolated Lateralized Overgrowth	1	36.6	1.9	3.2
69	ILO6	Isolated Lateralized Overgrowth	2	40.5	3.8	7.3
70	BWS16	Beckwith-Wiedemann	1	38.3	2.1	0.7
71	BWS16	Beckwith-Wiedemann	2	44.3	0.8	0.1
72	ILO7	Isolated Lateralized Overgrowth	1	6.2	43.0	41.2
73	ILO7	Isolated Lateralized Overgrowth	2	10.6	9.8	9.3
74	ILO7	Isolated Lateralized Overgrowth	3	15.8	3.1	3.1
75	BWS17	Beckwith-Wiedemann	1	94.6	5.8	3.3
76	BWS17	Beckwith-Wiedemann	2	99.0	5.5	4.4
77	BWS17	Beckwith-Wiedemann	3	102.7	3.5	0.5
78	BWS17	Beckwith-Wiedemann	4	111.3	4.3	5.8
79	BWS17	Beckwith-Wiedemann	5	115.7	4.2	4.9
80	BWS17	Beckwith-Wiedemann	6	106.4	3.9	0.1
81	BWS18	Beckwith-Wiedemann	1	35.5	1.1	0.3
82	BWS18	Beckwith-Wiedemann	2	39.5	1.0	0.2

83	BWS18	Beckwith-Wiedemann	3	47.6	0.9	1.3
84	BWS18	Beckwith-Wiedemann	4	49.4	0.8	0.8
85	BWS19	Beckwith-Wiedemann	1	5.6	73.0	94.5
86	BWS19	Beckwith-Wiedemann	2	9.3	20.0	17.2
87	BWS19	Beckwith-Wiedemann	3	12.5	11.4	9.8
88	BWS19	Beckwith-Wiedemann	4	16.0	6.2	4.3
89	BWS19	Beckwith-Wiedemann	5	19.5	8.6	9.2
90	BWS19	Beckwith-Wiedemann	6	22.8	7.9	9.6
91	ILO8	Isolated Lateralized Overgrowth	1	91.0	2.3	0.9
92	ILO9	Isolated Lateralized Overgrowth	1	128.8	3.1	0.8
93	ILO9	Isolated Lateralized Overgrowth	2	136.2	2.9	0.5
94	UO6	Undiagnosed Overgrowth	1	45.7	14.2	6.2
95	BWS20	Beckwith-Wiedemann	1	125.1	1.2	0.2
96	BWS21	Beckwith-Wiedemann	1	21.8	6.1	4.6
97	BWS22	Beckwith-Wiedemann	1	7.2	27.0	21.5
98	BWS22	Beckwith-Wiedemann	2	9.0	12.0	8.3
99	BWS22	Beckwith-Wiedemann	3	18.6	5.2	5.2
100	BWS23	Beckwith-Wiedemann	1	37.1	3.9	3.2
101	BWS23	Beckwith-Wiedemann	2	49.7	2.8	1.0
102	BWS23	Beckwith-Wiedemann	3	55.4	3.4	3.3
103	BWS24	Beckwith-Wiedemann	1	3.0	609.0	610.5
104	BWS25	Beckwith-Wiedemann	1	31.6	3.2	1.1
105	BWS25	Beckwith-Wiedemann	2	164.1	1.3	2.0
106	BWS26	Beckwith-Wiedemann	1	21.2	9.1	6.8
107	BWS26	Beckwith-Wiedemann	2	25.6	3.1	1.6
108	BWS26	Beckwith-Wiedemann	3	29.6	4.9	1.6
109	BWS26	Beckwith-Wiedemann	4	33.5	2.2	1.2
110	BWS26	Beckwith-Wiedemann	5	38.9	1.6	1.0
111	BWS26	Beckwith-Wiedemann	6	42.3	2.3	2.1
112	BWS27	Beckwith-Wiedemann	1	3.9	225.0	212.0
113	BWS28	Beckwith-Wiedemann	1	153.4	1.5	0.4

114	BWS29	Beckwith-Wiedemann	1	57.2	1.4	0.4
115	BWS30	Beckwith-Wiedemann	1	11.5	18.5	20.0
116	BWS30	Beckwith-Wiedemann	2	13.0	7.9	6.2
117	C1	Control	1	8.4	7.0	1.4
118	C2	Control	1	226.0	2.4	0.8
119	C3	Control	1	6.1	27.9	33.2
120	C4	Control	1	150.5	2.3	0.6
121	C5	Control	1	148.4	1.3	0.1
122	C5	Control	2	148.4	2.3	0.1
123	C6	Control	1	5.9	51.9	64.8
124	C7	Control	1	174.4	1.4	0.1
125	C8	Control	1	65.8	1.3	0.1
126	C9	Control	1	30.7	2.9	0.2
127	C10	Control	1	28.9	2.9	0.1
128	C10	Control	2	31.0	2.9	2.2
129	C11	Control	1	149.5	1.6	0.2
130	C12	Control	1	38.5	0.8	0.1
131	C13	Control	1	85.0	0.8	0.1
132	C14	Control	1	3.0	44.3	35.0
133	C15	Control	1	11.9	2.1	0.2
134	C16	Control	1	9.2	33.4	24.8
135	C17	Control	1	8.5	6.5	5.4
136	C18	Control	1	55.4	2.0	0.2
137	C19	Control	1	162.7	0.6	0.1
138	C20	Control	1	166.4	2.5	0.2
139	C21	Control	1	6.2	19.0	26.8
140	C22	Control	1	0.4	21318.0	20540.0
141	C23	Control	1	125.5	0.8	0.5
142	C24	Control	1	109.0	1.4	2.0
143	C25	Control	1	8.9	14.3	35.0
144	C26	Control	1	36.9	2.6	1.3

145	C27	Control	1	0.8	741.0	648.0
146	C28	Control	1	0.8	3386.0	3110.0
147	C28	Control	2	16.4	21.9	16.8
148	C29	Control	1	43.0	2.5	0.7
149	C30	Control	1	96.4	0.5	0.1
150	C31	Control	1	23.9	2.6	2.5
151	C32	Control	1	22.8	1.7	0.2
152	C33	Control	1	170.1	1.5	0.1
153	C34	Control	1	93.0	1.2	0.1
154	C34	Control	2	4.3	182.0	179.0
155	C35	Control	1	8.9	7.9	3.8
156	C36	Control	1	296.3	3.1	1.1
157	C37	Control	1	103.6	1.4	0.4
158	C38	Control	1	180.0	1.5	0.1
159	C39	Control	1	6.5	16.0	19.2
160	C40	Control	1	9.5	9.8	4.1
161	C41	Control	1	9.7	19.3	21.3
162	C42	Control	1	180.4	2.4	0.2
163	C43	Control	1	9.1	23.0	20.4
164	C44	Control	1	24.5	2.8	1.8
165	C44	Control	2	30.0	0.9	0.7
166	C45	Control	1	22.3	9.1	2.1
167	C46	Control	1	32.6	1.4	0.5
168	C47	Control	1	101.8	2.2	0.3
169	C48	Control	1	164.6	1.6	0.1
170	C49	Control	1	225.9	1.6	0.1
171	C50	Control	1	2.4	202.0	198.0
172	C50	Control	2	14.9	4.7	0.1
173	C51	Control	1	157.1	0.8	2.1
174	C51	Control	2	159.1	1.0	1.6
175	C52	Control	1	20.5	6.9	7.3

176	C52	Control	2	20.8	3.5	2.5
177	C53	Control	1	21.8	5.3	5.2
178	C54	Control	1	22.9	2.6	2.4
179	C55	Control	1	49.6	0.9	0.7
180	C56	Control	1	179.2	1.8	2.5
181	C57	Control	1	132.0	2.2	0.2
182	C58	Control	1	109.9	2.7	0.5
183	C59	Control	1	157.5	2.0	0.6
184	C60	Control	1	103.3	1.8	0.5
185	C61	Control	1	16.7	3.1	1.3
186	C61	Control	2	18.0	3.2	1.3
187	C62	Control	1	18.1	9.5	8.8
188	C63	Control	1	192.6	3.7	3.2
189	C64	Control	1	15.2	5.2	6.3
190	C64	Control	2	19.4	12.2	6.7
191	C65	Control	1	91.5	2.0	0.1
192	C66	Control	1	98.3	0.3	0.1
193	C67	Control	1	124.0	0.9	0.1
194	C68	Control	1	16.3	2.1	0.3
195	C68	Control	2	3.5	98.0	75.0
196	C69	Control	1	1.9	79.0	43.5
197	C69	Control	2	2.7	79.0	64.4
198	C70	Control	1	3.8	90.0	66.5
199	C71	Control	1	10.2	11.0	10.0
200	C72	Control	1	16.7	7.9	1.9
201	C73	Control	1	99.6	2.4	0.2
202	C74	Control	1	14.0	23.4	25.6
203	C75	Control	1	3.1		
204	C75	Control	2	8.1	9.4	5.9
205	C76	Control	1	151.9	6.6	0.7
206	C77	Control	1	7.1	8.6	8.5

207	C77	Control	2	8.9	6.2	0.6
208	C78	Control	1	2.7	182.0	245.0
209	C79	Control	1	9.2	21.3	13.2
210	C79	Control	2	18.9	5.6	3.8
211	C80	Control	1	171.6	3.1	0.1
212	C81	Control	1	161.1	4.8	0.2
213	C81	Control	1	220.1	1.2	0.4
214	C83	Control	1	252.1	2.1	0.3
215	C84	Control	1	19.2	5.3	6.2
216	C84	Control	2	30.6	3.0	2.1
217	C85	Control	1	29.5	3.2	0.1
218	C86	Control	1	29.9	3.1	1.0
219	C87	Control	1	146.9	2.0	0.1
220	C88	Control	1	98.9	2.1	0.8
221	C89	Control	1	41.1	4.5	3.5
222	C90	Control	1	23.9	1.2	0.2
223	C91	Control	1	149.3	1.7	0.1
224	C92	Control	1	0.9	6.2	2.9
225	C93	Control	1	66.7	2.1	0.1
226	C94	Control	1	156.7	1.1	0.4
227	C95	Control	1	8.8	10.0	5.5
228	C96	Control	1	15.9	3.2	0.1
229	C97	Control	1	104.5	1.0	0.1
230	C98	Control	1	89.0	2.8	0.9
231	C99	Control	1	31.3	2.4	0.1
232	C100	Control	1	16.7	1.5	0.3
233	C101	Control	1	3.2	9.9	14.3
234	C101	Control	2	14.1	4.1	2.7
235	C102	Control	1	130.5	2.1	0.1
236	C103	Control	1	117.9	1.6	0.2
237	C104	Control	1	102.7	1.5	0.1

238	C105	Control	1	30.3	1.7	0.1
239	C105	Control	2	31.2	1.4	1.6
240	C106	Control	1	66.5	2.1	2.1
241	C107	Control	1	228.5	2.1	0.3
242	C108	Control	1	174.3	2.3	2.4
243	C108	Control	2	181.7	2.3	0.2
244	C109	Control	1	109.6	2.1	0.8
245	C110	Control	1	94.3	1.1	1.5
246	C111	Control	1	96.6	1.0	1.6
247	C112	Control	1	4.5	186.0	178.0
248	C113	Control	1	10.0	7.2	5.1
249	C114	Control	1	13.6	10.5	14.5
250	C114	Control	2	17.3	4.3	5.5
251	C115	Control	1	29.0	5.6	1.5
252	C116	Control	1	52.4	0.8	0.1
253	C117	Control	1	151.7	5.1	6.8
254	C118	Control	1	91.8	0.9	0.2
255	C119	Control	1	29.8	1.3	0.5
256	C120	Control	1	70.2	0.5	3.1
257	C121	Control	1	57.1	1.5	2.7
258	C122	Control	1	142.7	1.5	0.5
259	C123	Control	1	27.5	1.0	0.2
